# Vasculoprotective Effects of Pomegranate (*Punica granatum* L.)

**DOI:** 10.3389/fphar.2018.00544

**Published:** 2018-05-24

**Authors:** Dongdong Wang, Cigdem Özen, Ibrahim M. Abu-Reidah, Sridevi Chigurupati, Jayanta Kumar Patra, Jarosław O. Horbanczuk, Artur Jóźwik, Nikolay T. Tzvetkov, Pavel Uhrin, Atanas G. Atanasov

**Affiliations:** ^1^Department of Molecular Biology, Institute of Genetics and Animal Breeding of the Polish Academy of Sciences, Jastrzebiec, Poland; ^2^Department of Pharmacognosy, Faculty of Life Sciences, University of Vienna, Vienna, Austria; ^3^Institute of Clinical Chemistry, University Hospital Zurich, University of Zurich, Zurich, Switzerland; ^4^Izmir International Biomedicine and Genome Institute, Dokuz Eylul University, Health Campus Balcova, Izmir, Turkey; ^5^Department of Chemistry, Faculty of Science, An-Najah National University, Nablus, Palestine; ^6^Department of Medicinal Chemistry and Pharmacognosy, College of Pharmacy, Qassim University, Buraidah, Saudi Arabia; ^7^Research Institute of Biotechnology and Medical Converged Science, Dongguk University-Seoul, Goyang, South Korea; ^8^Pharmaceutical Institute, University of Bonn, Bonn, Germany; ^9^Department of Molecular Design and Biochemical Pharmacology, Institute of Molecular Biology “Roumen Tsanev”, Bulgarian Academy of Sciences, Sofia, Bulgaria; ^10^Department of Vascular Biology and Thrombosis Research, Center for Physiology and Pharmacology, Medical University of Vienna, Vienna, Austria

**Keywords:** pomegranate, antioxidant, blood pressure, cardiovascular disease, vasculoprotective

## Abstract

Pomegranate (*Punica granatum* L.), one of the oldest known edible fruits, is nowadays broadly consumed throughout the world. Its fruits and seeds as well as other anatomical compartments (e.g., flowers and leaves) are rich in numerous bioactive compounds and therefore, the scientific interest in this plant has been constantly growing in recent years. It has been shown that pomegranate and its extracts exhibit potent antioxidative, antimicrobial, and anticarcinogenic properties. The present review summarizes some recent studies on pomegranate, highlighting mainly its vasculoprotective role attributed to the presence of hydrolyzable tannins ellagitannins and ellagic acid, as well as other compounds (e.g., anthocyanins and flavonoids). These *in vitro* and *in vivo* studies showed that substances derived from pomegranate reduce oxidative stress and platelet aggregation, diminish lipid uptake by macrophages, positively influence endothelial cell function, and are involved in blood pressure regulation. Clinical studies demonstrated that daily intake of pomegranate juice lessens hypertension and attenuates atherosclerosis in humans. Altogether, the reviewed studies point out the potential benefits of a broader use of pomegranate and its constituents as dietary supplements or as adjuvants in therapy of vascular diseases, such as hypertension, coronary artery disease, and peripheral artery disease.

## Introduction

Pomegranate (*Punica granatum* L.), belonging to *Punica* L. genus, *Punicaceae* family, is an ancient fruit native to Central Asia in regions spanning from Iran and Turkmenistan to northern India as well as in the Mediterranean area and the Middle East (Holland et al., 2009). Archaeologists have found carbonized pomegranate exocarps originated from the Early Bronze Age (3000 BC), e.g., in Jericho and from the Late Bronze Age in Cyprus (Ward, 2003; Boncuk, 2014). Pomegranate has been highly appreciated since centuries by different cultures. For example, in Ancient Egypt it was not only a part of the supply of fruits for pharaoh's residence (at around 1600 B.C.), but pomegranate was also painted on walls and tombs to symbolize life after death (Ward, 2003; Boncuk, 2014). Pomegranate used to play an important role in different religions, including Zoroastrianism, Judaism, Buddhism, Christianity, and Islam (Langley, 2000; Jurenka, 2008). It was praised, e.g., by the Old Testament of the Bible as “a sacred fruit conferring powers of fertility, abundance, and good luck” (Jurenka, 2008). Besides being a part of the mythology and consumed as a fruit, pomegranate has been known for its medical use. For example, the Ebers papyrus originating from about 1550 BC noted that the roots of the pomegranate tree were used to treat tapeworm parasites (Svenja, 2018). In addition, pomegranate was employed to treat diabetes by Indians (Saxena and Vikram, 2004) and to lessen tapeworm infestation also by Romans (Langley, 2000). The persisting significance of the medical use of pomegranate can be illustrated, for example, by the fact that in Great Britain the coats of arms of three royal colleague and the British Medical Association are decorated with the figure of this herb (Langley, 2000).

**Figure 1:**
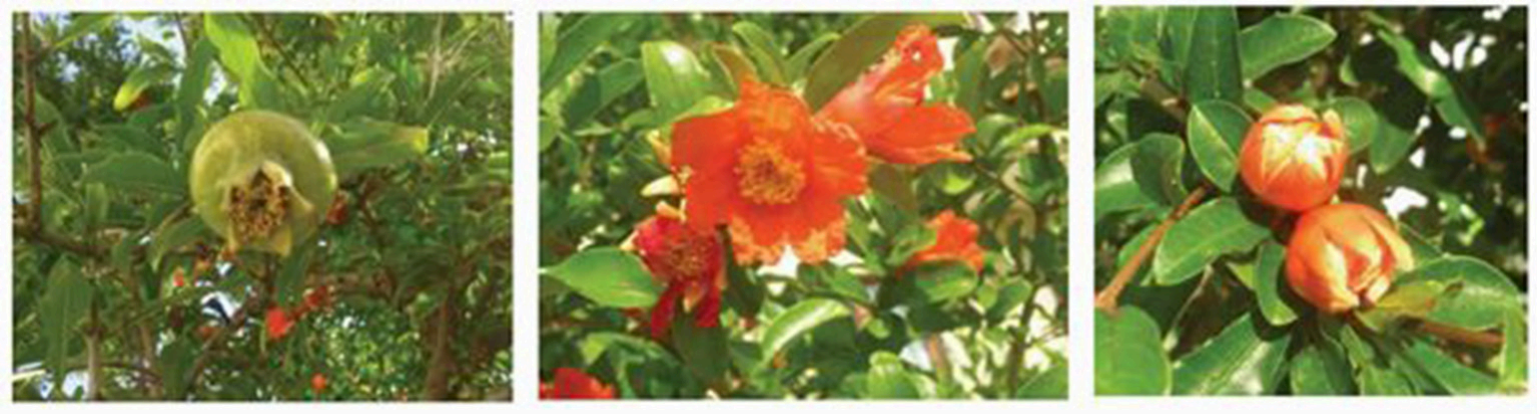
Fruits, flowers, and leaves of *Punica granatum* L.

Besides native regions spreading from Iran to northern India and the Mediterranean area and the Middle East, pomegranate is nowadays cultivated in subtropical Africa as well as in California, Arizona, and Mexico, as this plant requires high exposure to sunlight during summer and temperature not lower than ~12°C in winter (Levin, 2006; Holland et al., 2009). The pomegranate tree is about 2–3 m tall, glabrous, with multiple trunks and bushy appearance. The surface of the leaves is smooth and hairless, with a glossy appearance on the upper part of the leaf (Figure 1). The fruit ripens within 5–8 months after it has begun to form. During this process, the color of the external part of the fruit changes from yellow, green, or pink to fully red, pink, or deep purple (Figure 1). An edible juicy layer of a fruit varies in color from white to deep red (Holland et al., 2009).

Studies accomplished over the last several decades showed that pomegranate and its components exhibit potent antioxidative (Gil et al., 2000; Les et al., 2015), anti-inflammatory (Adams et al., 2006; Rasheed et al., 2009) as well as antibacterial, antimicrobial, and antifungal properties (Naz et al., 2007; Fawole et al., 2012; Elsherbiny et al., 2016; Wafa et al., 2017). In addition to these *in vitro* studies, *in vivo* and *in vitro* studies showed that pomegranate exhibits anti-hypertensive (Mohan et al., 2010; Dos Santos et al., 2016; Arun et al., 2017) and antiproliferative properties (Albrecht et al., 2004; Malik et al., 2005; Malik and Mukhtar, 2006). Pomegranate and its constituents have been tested for their use as adjuvant therapy for treatment of several forms of oncological diseases, mainly of prostate cancer (Lansky and Newman, 2007; Paller et al., 2013; Panth et al., 2017; Sharma et al., 2017). Furthermore, numerous pre-clinical studies have pointed out the beneficial effects of intake of pomegranate juice or pomegranate extracts in a variety of conditions. For example, such treatment improved sperm quality in mice (Türk et al., 2008), lowered amyloid deposition in a mouse model of Alzheimer's disease (Hartman et al., 2006), and lessened neuronal damage in a mouse neonatal hypoxic-ischemic brain injury model (Loren et al., 2005). In addition, single intraperitoneal injection with pomegranate extract applied to fishes that had been naturally infected with lymphocystis disease virus, stimulated their innate immune response, and reduced their mortality due to lymphocystis infection (Harikrishnan et al., 2010). In humans, oral administration of pomegranate extract enriched with ellagic acid is beneficial for minimizing ultraviolet-induced skin damage (Kasai et al., 2006), while hydro-alcoholic extracts of pomegranate have a significant antibacterial activity and are therefore useful for treatment of dental plaques (Menezes et al., 2009). Many studies also demonstrated potent vasculoprotective effects of pomegranate and its constituents, as presented below.

## Bioactive constituents of pomegranate

Bioactive substances of pomegranate include, for example, hydrolyzable tannins (gallotannins and ellagitannins), ellagic acid and its derivatives, gallic acid, anthocyanins/anthocyanidins, proanthocyanidins, flavonoids, vitamins, as well as sterols, lignans, saccharides, fatty acids, organic acids, terpenes, and terpenoids, among others. Ellagitannins and gallotannins together with ellagic acid and its derivatives are crucial bioactive compounds of pomegranate (Amakura et al., 2000a; Gil et al., 2000; Fischer et al., 2011a; Borges and Crozier, 2012; Brighenti et al., 2017). Furthermore, ellagitannins and gallotannins are hydrolyzed to ellagic acid and glucose or gallic acid and glucose, respectively (Arapitsas, 2012). In addition, pomegranate is a source of numerous (poly)phenolic compounds (Fischer et al., 2011a). Anthocyanins present in pomegranate comprise mainly delphinidin 3-glucoside, delphinidin 3,5-diglucoside, pelargonidin 3-glucoside, pelargonidin 3,5-diglucoside, cyanidin 3-glucoside, and cyanidin 3,5-diglucoside (Alighourchi et al., 2008; Fischer et al., 2013; Lantzouraki et al., 2015), and the characteristic colors of pomegranate fruits are attributed to them. Pomegranate seeds contain different fatty acids with the most represented punicic acid (Schubert et al., 1999; Kaufman and Wiesman, 2007; Pande and Akoh, 2009; Verardo et al., 2014; Górnaś and Rudzinska, 2016). Flavol-3-ols, flavonoid glycosides, phenolic acids, and hydrolyzable tannins represent main phenolic compounds in pomegranate seed residue (He et al., 2011). In pomegranate peel, gallic acid is a major phenolic constituent while kaempferol-3-*O*-glucoside is the most represented flavonoid (Ambigaipalan et al., 2016). Triterpenoids oleanolic acid and ursolic acid are present in pomegranate flower (Fu et al., 2014). Volatile components of pomegranate comprise monoterpenes, monoterpenoids, aldehydes, alcohols, and linear hydrocarbons monoterpenes, especially represented by alpha-terpinene, alpha-terpineol, and 3-carene (Vázquez-Araújo et al., 2011; Carbonell-Barrachina et al., 2012). An overview of compounds identified in pomegranate is outlined in Table 1.

**Table 1:** List of compounds identified in pomegranate (*Punica granatum* L.).

**Pomegranate phytochemicals**	**Pomegranate part**	**References**
**(1) ALKALOIDS**		
Caffeine	Peel^*^	Elsherbiny et al., 2016
*N*-(2′,5′-dihydroxyphenyl) pyridium chloride	Leaf	Nawwar et al., 1994b
Peelletierine	Peel, bark	Neuhofer et al., 1993; Vidal et al., 2003
**(2) ANTHOCYANINS/ANTHOCYANIDINS**		
Cyanidin glucosides and derivatives	Juice, seed, peel	Hernandez et al., 1999; Noda et al., 2002; Alighourchi et al., 2008; Türkyilmaz, 2013; Ambigaipalan et al., 2016; Wafa et al., 2017
Delphinidin glucosides and derivatives	Juice, peel	Hernandez et al., 1999; Noda et al., 2002; Alighourchi et al., 2008; Borges and Crozier, 2012; Türkyilmaz, 2013; Ambigaipalan et al., 2016; Wafa et al., 2017
(Epi) afzelchin-delphinidin-3-O-hexoside	Seed	Ambigaipalan et al., 2017
Malvidin glucosides and derivatives	Juice	Borges and Crozier, 2012; Pérez-Ramírez et al., 2018
Pelargonidin glucosides and derivatives	Juice, peel	Hernandez et al., 1999; Noda et al., 2002; Alighourchi et al., 2008; Türkyilmaz, 2013; Wafa et al., 2017
Peonidin-3-*O*-(6”-*O*-acetyl)glucoside	Juice	Borges and Crozier, 2012
Vitisin A	Juice	Borges and Crozier, 2012
**(3) ELLAGIC ACID AND DERIVATIVES**		
Ellagic acid	Juice, peel, seed, flower	Amakura et al., 2000b; Gil et al., 2000; Wang et al., 2004; Jain et al., 2011; Wafa et al., 2017
Ellagic acid glucosides and derivatives	Juice, peel	Gil et al., 2000; Wafa et al., 2017
**(4) FATTY ACIDS**		
Arachidic acid, behenic acid, docosadienoic acid, eicosapentaenoic acid, erucic acid, gondoic acid, lignoceric acid, linoleic acid, linolelaidic acid, linolenic acid, myristic acid, margaric acid, nervonic acid, oleic acid, palmitic acid, palmitoleic acid, punicic acid, stearic acid, *cis*-vaccenic acid	Seed	Hopkins and Chisholm, 1968; Schubert et al., 1999; Kaufman and Wiesman, 2007; Pande and Akoh, 2009; Elfalleh et al., 2011; Verardo et al., 2014; Siano et al., 2016
**(5) FLAVONOIDS AND DERIVATIVES**		
Acetyl prunin, diosmetin glucoside	Juice	Fanali et al., 2016
Apigenine	Leaf	Nawwar et al., 1994b
Apigenin-rhamnoside, chrysin	Juice	Lantzouraki et al., 2015
Catechin	Juice, seed, peel	De Pascual-Teresa et al., 2000; Mphahlele et al., 2014; Ambigaipalan et al., 2016
Datiscetin-hexoside	Juice	Mena et al., 2012
Dihydroxygallocatechin	Peel	Ambigaipalan et al., 2016
Epicatechin	Juice, peel	De Pascual-Teresa et al., 2000; Mphahlele et al., 2014
Eriodictyol 7-*O*-β-glucoside	Juice	Mphahlele et al., 2014
Flavan-3-ol	Juice, peel	De Pascual-Teresa et al., 2000
Gallocatechin	Peel	Wafa et al., 2017
Hesperidin	Juice	Mphahlele et al., 2014
Kaempferol	Peel	Van Elswijk et al., 2004
Kaempferol glucoside(s)	Juice, seed, peel	Van Elswijk et al., 2004; Mphahlele et al., 2014; Lantzouraki et al., 2015; Ambigaipalan et al., 2016
Luteolin	Peel, fruit	Van Elswijk et al., 2004; Han et al., 2015
Myricetin and its glucoside	Juice	Naz et al., 2007; Lantzouraki et al., 2015
Naringin	Juice, peel	Kim et al., 2002; Mphahlele et al., 2014
Phloretin	Peel, seed, juice	Han et al., 2015
Phloridzin	Juice	Hmid et al., 2017
Pinocembrin	Juice	Calani et al., 2013
Quercetin and its derivatives	Juice, seed, peel	Artik, 1998; Naz et al., 2007; Borges and Crozier, 2012; Han et al., 2015; Lantzouraki et al., 2015; Ambigaipalan et al., 2016
Rutin	Juice, peel	Artik, 1998; Mphahlele et al., 2014
Taxifolin and its glycosides	Peel, seed, juice	Calani et al., 2013; Han et al., 2015
**(6) LIGNANS**		
Isolariciresinol, matairesinol, medioresinol, pinoresinol, secoisolariciresinol, syringaresinol	Fruit, seed	Bonzanini et al., 2009
**(7) ORGANIC ACIDS**		
Citric acid	Juice	Poyrazoglu et al., 2002; Carbonell-Barrachina et al., 2012; Gundogdu and Yilmaz, 2012; Legua et al., 2012; Lantzouraki et al., 2015
Fumaric acid	Juice	Poyrazoglu et al., 2002; Gundogdu and Yilmaz, 2012
Lactic acid	Juice	Gundogdu and Yilmaz, 2012
Malic acid	Juice	Poyrazoglu et al., 2002; Carbonell-Barrachina et al., 2012; Lantzouraki et al., 2015
Methylmalonic acid	Juice	Alper et al., 2011
Oxalic acid	Juice	Legua et al., 2012
Quinic acid	Juice, peel	Artik, 1998; Amakura et al., 2000a; Ehling and Cole, 2011
Succinic acid	Juice	Poyrazoglu et al., 2002; Alper et al., 2011
Tartaric acid	Juice	Poyrazoglu et al., 2002; Ehling and Cole, 2011; Legua et al., 2012
Uronic acid	Peel	Hasnaoui et al., 2014
**(8) OTHER PHENOLIC COMPOUNDS**		
3-Hydroxytyrosol	Peel	Elsherbiny et al., 2016
Benzaldehyde	Peel	Hadrich et al., 2014
Benzoic acid	Peel	Hadrich et al., 2014
Brevifolin carboxylic acid	Fruit, juice	Fischer et al., 2011a,b
Caffeic acid and its hexoside	Juice, peel	Artik, 1998; Amakura et al., 2000a; Lantzouraki et al., 2015
Chlorogenic acid	Juice, peel	Artik, 1998; Amakura et al., 2000a; Hasnaoui et al., 2014
Cinnamic acid	Juice	Lantzouraki et al., 2015
Coniferyl 9-*O*-[β-d-apiofuranosyl (1 → 6)]-*O*-β-d-glucopyranoside	Seed	Wang et al., 2004
Cyanidin-pentoside-hexoside	Fruit	Fischer et al., 2011a
Ethyl cinnamate	Juice	Cadwallader et al., 2010
Ferulic acid and its hexoside	Juice	Lantzouraki et al., 2015
Gallic acid	Juice, seed, peel	Amakura et al., 2000b; Huang et al., 2005a; Jain et al., 2011; Mphahlele et al., 2014; Ambigaipalan et al., 2016; Fanali et al., 2016
Protocatechuic acid	Juice, seed, peel	Ambigaipalan et al., 2016; Fanali et al., 2016
*p*-Coumaric acid	Juice, peel, seed	Artik, 1998; Amakura et al., 2000a; Ambigaipalan et al., 2017
Salycilic acid	Peel	Elsherbiny et al., 2016
Sesamin, 4-hydroxysesamin	Peel	Jiang et al., 2012
Vanillic acid	Juice	Mena et al., 2012
**(9) PROANTHOCYANIDINS**		
Procyanidin dimer B2 and B3	Peel	Ambigaipalan et al., 2016
Arabinose, xylose, galactose, glucose, mannose, rhamnose	Peel	Hasnaoui et al., 2014
**(10) SACCHARIDES**		
Glucose, fructose, maltose, sucrose	Juice	Carbonell-Barrachina et al., 2012; Legua et al., 2012; Vegara et al., 2014; Conidi et al., 2017
**(11) STEROLS**		
β-Sitosteryl acetate	Peel	Jiang et al., 2012
Avenasterol, Δ7-avenasterol, campesterol, cycloartenol, Δ7-stigmasterol, stigmasterol, β-sitosterol	Seed	Górnaś and Rudzinska, 2016
Camesterol	Seed	Abd El Wahab et al., 1998
Daucosterol	Seed	Wang et al., 2004
Stigmasterol	Seed	Abd El Wahab et al., 1998
**(12) TANNINS (GALLOTANNINS AND ELLAGITANNINS AND THEIR DERIVATIVES)**		
1,2,3-Tri-*O*-galloyl-β-^4^C1-glucose	Leaf	Nawwar et al., 1994a
2-*O*-Galloylpunicalin	Juice	Borges and Crozier, 2012
3,3′-Di-*O*-methylellagic acid	Seed	Wang et al., 2004
3,3′,4′-Tri-O-methylellagic acid	Seed	Wang et al., 2004
Castalagin	Juice, peel	Fischer et al., 2011a
Castalin	Fruit, juice	Fischer et al., 2011b
Casuarinin (Galloyl-bis-HHDP-hexoside)	Peel	Satomi et al., 1993; Ambigaipalan et al., 2016
Corilagin (Galloyl-HHDP-hexoside)	Peel, leaf	Satomi et al., 1993; Nawwar et al., 1994a; Ambigaipalan et al., 2016
Epicatechin gallate	Peel	Ambigaipalan et al., 2016
Flavogallonic acid	Peel	Jiang et al., 2012
Gallagic acid	Peel, juice	Tzulker et al., 2007
Gallagyldilacton	Peel	Satomi et al., 1993; Anibal et al., 2013
Granatin A/B	Peel	Tanaka et al., 1990; Wafa et al., 2017
Lagerstannin C (Galloyl-HHDP-gluconic)	Peel	Wafa et al., 2017
Pedunculagin I (bis-HHDP-hexoside)	Juice, peel	Satomi et al., 1993; Lantzouraki et al., 2015; Wafa et al., 2017
Pentagalloylglucopyranose	Seed	He et al., 2011
Punicacortein A, B, C, and D	Peel, bark	Tanaka et al., 1986a; Anibal et al., 2013
Punicafolin	Leaf	Nawwar et al., 1994a
Punicalagin (HHDP-gallagyl-hexoside)	Juice, peel, leaf	Tanaka et al., 1986b; Jain et al., 2011; Anibal et al., 2013; Lantzouraki et al., 2015
Punicalin α and β	Peel, juice, leaf	Tanaka et al., 1986b; Tzulker et al., 2007; Jain et al., 2011; Wafa et al., 2017
Punicatannin C	Flower	Yuan et al., 2013
Punigluconin (Digalloyl-HHDP-glucoside)	Peel	Wafa et al., 2017
Tellimagrandin	Peel	Satomi et al., 1993
Tergallagic acid-*O*-glucoside	Juice	Borges and Crozier, 2012
Valoneic acid bilactone	Juice	Fischer et al., 2011a,b
**(13) TERPENES AND TERPENOIDS**		
3-Carene, α-terpinene, α-terpineol, eugenol	Juice	Carbonell-Barrachina et al., 2012
Asiatic acid	Flower	Batta and Rangaswami, 1973
Betulinol, 24-methylenecycloartenol, cycloartenol, squalene	Seed	Verardo et al., 2014
Camphor	Peel	Hadrich et al., 2014
Eugenol	Juice	Carbonell-Barrachina et al., 2012
Maslinic acid	Flower	Batta and Rangaswami, 1973
Oleanolic acid	Flower	Huang et al., 2005b; Fu et al., 2014
α/β-Pinene, limonene, terpineol, β-farnesene, β-caryophyllene, bisabolene	Juice	Vázquez-Araújo et al., 2011
Punicaone, 1β-hydroxy-3-oxoolean-12-en-28-oic acid, 3β,24-dihydroxyurs-12-en-28-oic acid, betulin, betulinic acid, borneol, friedelin, lantanolic acid, lupeol, oleanic acid	Peel	Jiang et al., 2012
Ursolic acid	Seed, flower	Ahmed et al., 1995; Huang et al., 2005a; Fu et al., 2014
**(14) VITAMINS**		
Vitamin C	Juice	Dumlu and Gurkan, 2007
**(15) XANTONOIDS**		
Mangiferin	Peel	Elsherbiny et al., 2016

* *Peel (pericarp, rind, and hull are synonyms)*.

## Vasculoprotective effects of various parts of pomegranate revealed in *in vitro* and *in vivo* models

Many pomegranate-derived compounds exhibit a wide range of vasculoprotective effects. Various pomegranate parts (components) have proven to reduce oxidative stress, lipid peroxidation, and generation of foam cells, to positively influence endothelial cell function (by increasing NO levels and lowering glucose levels), to attenuate platelet aggregation and diminish hypertension, thus altogether improving vascular function, as presented below. In addition, pomegranate and its components are protective against toxicity induced by chemicals or drugs (Table 2 and the text below).

**Table 2:** Vasculoprotective effects of pomegranate determined in *in vitro* and *in vivo* pre-clinical studies.

**Vasculoprotective effects**	**Pomegranate part**	**References**
Antioxidative properties *in vitro* and *in vivo*	Juice, fruit extract, peel extract	Gil et al., 2000; Les et al., 2015; Delgado et al., 2016
Suppression of peroxidation of plasma lipids, induction of serum paraoxonase activity, lowering lipid uptake by macrophages, and diminishing development of atherosclerosis in mice	Juice, fruit extract	Aviram et al., 2000; Fuhrman et al., 2005; Rosenblat et al., 2015; Mollazadeh et al., 2016
Improvement of endothelial cell function *in vitro*, in mice and pigs [due to an activation of the protein kinase B (Akt)/eNOS pathway, an inhibition of a superoxide anion-mediated disappearance of NO, and reduction of vascular inflammation]	Juice, fruit extract	De Nigris et al., 2005; de Nigris et al., 2006; De Nigris et al., 2007; Ignarro et al., 2006; Vilahur et al., 2015
Reduction the collagen- and arachidonic acid-induced platelet aggregation *ex vivo*	Juice, fruit extract	Aviram et al., 2000; Mattiello et al., 2009
Reduction in activity of angiotensin-converting enzyme (ACE); decrease in mean arterial blood pressure in rats	Juice, fruit extract, peel extract	Mohan et al., 2010; Dos Santos et al., 2016; Arun et al., 2017
Lessening cardiac toxicity induced by drugs or smoking (diminishing lipid peroxidation and increasing levels of antioxidant enzymes)	Juice, fruit extract	Jadeja et al., 2010; Al Hariri et al., 2016
Reduction of blood glucose levels in a variety of mouse and rat models (effects mediated via upregulation of PPAR-γ leading to an increase in insulin sensitivity)	Seed-, flower-, and peel-extract	Das et al., 2001; Huang et al., 2005a; Li et al., 2005; Vroegrijk et al., 2011; Salwe et al., 2015
Lowering fatty acid, triglycerides and total cholesterol plasma levels as well as cardiac triglycerides (in Zucker diabetic fatty rats)	Flower extract	Huang et al., 2005b

### Pomegranate juice and extract

In 2000s, Gil et al. in their pivotal study pointed out the strong antioxidant properties of pomegranate juice enriched with tannin punicalagin, anthocyanins, ellagic acid derivatives, as well as other phenolic substances. Using different analytical methods, the authors revealed potent antioxidant activities of pomegranate juice that were three times higher than the well-known antioxidative properties of red wine or green tea (Gil et al., 2000). These findings were confirmed by subsequent studies that additionally pointed to vasculoprotective effects of pomegranate products, as presented below.

In a study involving mice as well as human volunteers, pomegranate juice intake attenuated oxidative stress (Aviram et al., 2000). More specifically, in apolipoprotein E-deficient mice, food supplementation with pomegranate juice reduced by 44% the size of atherosclerotic lesions and diminished the number of foam cells in such lesions (Aviram et al., 2000). In humans, intake of pomegranate juice diminished the susceptibility of low-density lipoproteins (LDLs) to aggregate and enhanced by up to 20% the activity of serum paraoxonase (Aviram et al., 2000), an esterase that is associated with high-density lipoproteins (HDLs) and can protect lipids against peroxidation (Chistiakov et al., 2017). Pomegranate juice also inhibited the oxidized LDL (oxLDL) uptake and cholesterol biosynthesis in a J774.A1 macrophage-like cell line (Fuhrman et al., 2005). A study utilizing diabetic mice model suggested that these protective effects might be due to the presence of unique complex sugars and/or phenolic sugars in pomegranate juice (Rozenberg et al., 2006). Another study involved high and low exercise lifestyle mimicking rats (high- and low-capacity runners) fed with pomegranate juice for 3 weeks (Rosenblat et al., 2015). While the effects were stronger in a group of low-capacity runners, the consumption of pomegranate juice decreased the cellular oxidation and increased the paraoxonase 2 activity in peritoneal macrophages from both animal groups when compared with non-treated cohorts of animals (Rosenblat et al., 2015).

In cultured human coronary artery endothelial cells exposed to high shear stress, pomegranate juice down-regulated the expression of redox sensitive genes ELK-1 and p-JUN and increased the expression of endothelial nitric oxide synthase (eNOS) (De Nigris et al., 2005) that is necessary for the proper functioning of blood endothelial cells (Vallance and Chan, 2001). In addition, an intake of pomegranate juice by LDL receptor-deficient mice fed with high-cholesterol diet, lessened progression of atherogenesis at different stages of the disease (De Nigris et al., 2005). Another *in vitro* study using cultured bovine pulmonary artery endothelial cells showed that the presence of even very low amounts of pomegranate juice in the cultivation medium protects the generated nitric oxide (NO) against its oxidative destruction (via an inhibition of a superoxide anion-mediated disappearance of NO, leading to an enhancement of the bioavailability of NO) (Ignarro et al., 2006). Another study of this group showed that presence of pomegranate juice in human coronary artery endothelial cells reverts down-regulation of the expression of eNOS caused by the addition of oxLDL (de Nigris et al., 2006). In a study accomplished in hypercholesterolemic mice, an intake of pomegranate juice enriched with punicalagin increased the eNOS expression and decelerated the progression of atherosclerosis, as well as enhanced nitrates levels (De Nigris et al., 2007). In pigs, an intake of the commercial pomegranate extract Pomanox® made from dried pomegranate skin or husk could reduce coronary endothelial dysfunction induced by hyperlipidemia (Vilahur et al., 2015). These beneficial effects included an activation of the protein kinase B (Akt)/eNOS pathway and an attenuation of vascular inflammation as well as of vascular damage induced by oxidative stress (Vilahur et al., 2015).

Furthermore, pomegranate juice attenuated the aggregation of human platelets exposed to collagen or arachidonic acid *ex vivo* (Aviram et al., 2000; Mattiello et al., 2009), by attenuating calcium mobilization, thromboxane A2 production, and hydrogen peroxide formation (Mattiello et al., 2009). These effects were assigned to the presence of polyphenols in pomegranate products (Mattiello et al., 2009). It was also shown that pomegranate fruit extract was active at a 2.0 μM concentration that is possible to be achieved after polyphenol-rich food intake by humans (Mattiello et al., 2009). On the other side, pomegranate seed oil inhibited cyclooxygenase (COX) (Schubert et al., 1999), the key enzyme catalyzing the conversion of arachidonic acid to prostaglandin (PGI2) (Grosser et al., 2006). The latter substance is known as a potent vasoprotective factor inhibiting platelet adhesion and thrombus formation on endothelium (Weiss and Turitto, 1979). In addition, feeding of rats with pomegranate extract diminished in colonic mucosa levels of COX-2, prostaglandin E2 (PGE2) as well as inducible nitric oxide synthase (iNOS) (Larrosa et al., 2010b).

Some other works investigated how pomegranate affects arterial hypertension, an important risk factor for cardiovascular diseases (Pickering, 1972). For example, in a study involved the use of Wistar rats in which diabetes was induced by streptozotocin administration, and the animals were additionally challenged by a subcutaneous administration of angiotensin II to induce hypertension, a prolonged administration of pomegranate juice (for 4 weeks) reduced activity of angiotensin converting enzyme (ACE), as well as decreased mean arterial blood pressure in comparison with non-treated animals (Mohan et al., 2010).

Pomegranate fruit extracts were also studied regarding their protective effect against cardiac toxicity induced by drugs or smoking. For example, detrimental effects of a cardiotoxic drug isoproterenol (known to cause a cardiac necrosis leading to a myocardial infarction) were reduced upon pre-treatment of rats with pomegranate juice for 30 consecutive days before isoproterenol treatment (Jadeja et al., 2010). Such pre-treatment significantly lessened an increase in the heart weight, infarction size, plasma marker enzymes, lipid peroxidation levels as well as levels of Ca^2+^ ATPase (Jadeja et al., 2010). The protective effects of pomegranate juice intake were also demonstrated in a study using rats in which a cardiac hypertrophy was induced by cigarette smoke exposure (Al Hariri et al., 2016).

### Pomegranate seed oil

Pomegranate seeds comprise about 3% of the pomegranate weight and contain about 12–20% seed oil (Lansky and Newman, 2007) that is rich in fatty acids and contains mainly punicic acid (Kaufman and Wiesman, 2007; Verardo et al., 2014; Górnaś and Rudzinska, 2016).

In rats with streptozotocin-induced diabetes, oral feeding with seed extracts significantly reduced blood glucose levels (Das et al., 2001). In mice, an intake of pomegranate seed oil counteracted their obesity induced by a high-fat diet by enhancing peripheral insulin sensitivity (Vroegrijk et al., 2011). Oral treatment of the above cited diabetic rats with pomegranate seed oil significantly decreased peroxidation of plasma lipids (Mollazadeh et al., 2016). In addition, such treatment diminished malondialdehyde content in homogenates from the heart and kidney tissues, and reduced triglyceride levels in treated animals in comparison to the control cohort (Mollazadeh et al., 2016).

### Pomegranate flower, peel, and leaf extracts

A 6-week oral administration of pomegranate flower extracts suppressed plasma glucose levels in Zucker diabetic fatty rats following their exposure to glucose-loading. In addition, such treatment in these animals increased cardiac peroxisome proliferator-activated receptor gamma (PPAR-γ) mRNA expression as well as restored the down-regulated cardiac glucose transporter (GLUT)-4 mRNA, altogether improving insulin sensitivity (Huang et al., 2005a). These beneficial effects were assigned mainly to the presence of gallic acid (Huang et al., 2005a). A long-term treatment of Zucker diabetic fatty rats with pomegranate flower extracts was cardioprotective, as it lowered their fatty acid-, triglycerides-, and total cholesterol plasma levels as well as reduced the cardiac triglycerides content (Huang et al., 2005b). In another study, oral administration of pomegranate flower extracts decreased plasma glucose levels in non-fasted diabetic rats (but not in fasted-diabetic rats or in normal rats). This study also showed that pomegranate flower extracts inhibit α-glucosidase (a key enzyme for carbohydrate digestion in intestines) and administration of pomegranate flower extracts may improve postprandial hyperglycemia in type 2 diabetes, and altogether diminish the risk of cardiovascular dysfunctions (Li et al., 2005). In mice fed with a high-fat diet to induce obesity, treatment with pomegranate leaf extract decreased body weight, energy intake as well as total cholesterol, triglyceride, and glucose levels (Lei et al., 2007). Administration of hydroalcoholic peel or leaf extracts of pomegranate for 28 days decreased blood glucose levels in a Wister rat model of diabetes induced by streptozotocin (Salwe et al., 2015). Hydroalcoholic peel extracts of pomegranate were also tested in spontaneously hypertensive ovariectomized female rats (an animal model for menopause characterized by an increase in the superoxide anion levels; Delgado et al., 2016). Such treatment diminished elevation of superoxide anion levels and lessened oxidative stress in this animal model (Delgado et al., 2016). Treatment of spontaneously hypertensive rats of different ages for 30 days with pomegranate peel extracts, significantly reduced systolic blood pressure, ACE activity, oxidative stress as well as vascular remodeling (Dos Santos et al., 2016). A recent *in vitro* study showed that pomegranate peel methanolic extracts potently scavenge superoxide and hydroxyl radicals, protect LDL against oxidation and suppress ACE activity (Arun et al., 2017). Altogether, these studies demonstrated that also the non-edible parts of pomegranate—peel and leaves—exhibit vasculoprotective effects.

## Vasculoprotective effects of pure compounds derived from pomegranate

Studies presented above showed the numerous vasculoprotective effects of different parts of the pomegranate. It was suggested that many of these protective effects are caused by the presence of hydrolyzable tannins (ellagitannins and gallotannins), their derivative ellagic acid, or their common metabolites urolithins (Table 3 and the text below).

**Table 3:** Vasculoprotective effects of pomegranate-derived substances or their metabolites, as determined *in vitro* and *in vivo* pre-clinical studies.

**Vasculoprotective effects**	**Vasculoprotective substances**	**References**
Induction of paraoxonase 2 and reduction in oxidative stress in isolated macrophages	Punicalagin, gallic acid	Shiner et al., 2007
Attenuation of reactive oxygen species (ROS) generation and prevention of eNOS downregulation induced by oxLDL in HUVECs. Stimulation of vasorelaxation of the rat thoracic aorta *ex vivo*, via an endothelium-dependent mechanism and through an inhibition of calcium influx	Ellagic acid	Lee et al., 2010; Ou et al., 2010; Yilmaz and Usta, 2013
Suppression of formation of advanced glycation end products (AGEs) *in vitro* and in mice	Punicalin, punicalagin, ellagic acid, gallic acid	Kumagai et al., 2015
Inhibition of lipid metabolism in adipocytes	Punicalagin, ellagic acid	Les et al., 2017
Antioxidative properties in a cell-based assay *in vitro*	Urolithins	Bialonska et al., 2009
Inhibition of adhesion of monocytes to endothelial cells, of secretion of a cellular adhesion molecule (VCAM-1) and pro-inflammatory cytokine (IL-6). Decrease in the accumulation of cholesterol in THP-1-derived macrophages	Ellagic acid, urolithin A glucuronide, other urolithins	Gimenez-Bastida et al., 2012; Mele et al., 2016
Attenuation of endothelial dysfunction induced by oxLDL in cultured human artery endothelial cells, partly by counteracting eNOS-dependent decrease in NO production. Reduction in myocardial ischemia/reperfusion injury and myocardial infarct size *in vivo*	Urolithin A	Han et al., 2016; Tang et al., 2017
Anti-hypertensive effects of sweetie juice in humans	Naringin	Reshef et al., 2005
Amelioration of glucose tolerance and diminishing obesity-related inflammation via activation of PPAR-γ and -α	Puninic acid	Hontecillas et al., 2009

Pomegranate ellagitannins and a single high molecular weight ellagitannin punicalagin, attenuated the inflammatory cell signaling in colon cancer cells (Adams et al., 2006). Punicalagin and gallic acid induced in isolated macrophages the expression of paraoxonase 2 (Shiner et al., 2007). These substances also reduced oxidative stress in macrophages via activation of transcription factors PPAR-γ and activator protein 1 (AP-1; Shiner et al., 2007).

Single components (e.g., punicalin, punicalagin, ellagic acid, and gallic acid) isolated from pomegranate fruit suppressed the formation of advanced glycation end products (AGEs, known to contribute to a number of diseases including diabetic complications and arteriosclerosis) from bovine serum albumin and sugar in antiglycation assays *in vitro* (Kumagai et al., 2015). Pomegranate fruit extracts also reduced the accumulation of AGEs in mice fed with a high-fat and high-sucrose diet (Kumagai et al., 2015). In addition, punicalagin and ellagic acid inhibited lipid metabolism in mouse and human adipocytes *ex vivo* (Les et al., 2017).

Effects of ellagic acid on reactive oxygen species (ROS) generation were also investigated in endothelial cells. Pre-treatment of HUVECs with ellagic acid attenuated ROS production and prevented eNOS downregulation induced by oxLDL (Lee et al., 2010; Ou et al., 2010). *Ex vivo*, ellagic acid stimulated vasorelaxation of the rat thoracic aorta via an endothelium-dependent mechanism and an inhibition of calcium influx (Yilmaz and Usta, 2013). Nevertheless, as ellagitannins and ellagic acid *in vivo* metabolize into urolithins that enter systemic circulation (Cerdáet al., 2005; Larrosa et al., 2010a), researchers also studied how these metabolites affect the vascular function.

The antioxidant properties of different urolithins were evaluated in a cell-based assay and the results showed that urolithin C and D were more potent antioxidants than the parental substance ellagic acid and punicalagin (Bialonska et al., 2009). Nonetheless, although an *in vitro* antioxidant potential of urolithin A was relatively low in comparison with other urolithins, its plasma concentrations was the highest among them (Bialonska et al., 2009). In a subsequent study, urolithin A glucuronide inhibited adhesion of monocytes to endothelial cells in the micromolar range (5–15 μM), suggesting that the beneficial effects of pomegranate intake on the vasculature might be partly mediated by urolithin A glucuronide (Gimenez-Bastida et al., 2012). In addition, a recent *in vitro* study showed potent anti-atherogenic properties of ellagic acid and some urolithins (Mele et al., 2016). All these compounds reduced the adhesion of THP-1 derived macrophages to HUVECs and diminished secretion of soluble vascular cell adhesion molecule-1 (VCAM-1) and inflammatory interleukin-6 (IL-6) (Mele et al., 2016). In a study utilizing cultured human artery endothelial cells, urolithin A attenuated endothelial dysfunction induced by oxLDL (Han et al., 2016). These effects were partly mediated by counteracting eNOS-dependent decrease in NO production (Han et al., 2016). In addition, urolithin A reduced the expression of intracellular adhesion molecule-1 (ICAM-1) and monocyte chemotactic protein 1 (MCP-1), upon adhesion of THP-1 cells to the endothelial cells. Urolithin A also suppressed the expression of tumor necrosis factor-α (TNF-α), IL-6 and endothelin-1, increased PPAR-γ mRNA expression, and downregulated phosphorylation of the extracellular signal-regulated protein kinase 1/2 (ERK1/2) (Han et al., 2016). In another study, urolithin A inhibited heme peroxidases [myeloperoxidase (MPO) and lactoperoxidase (LPO)] more effectively than its parent compound ellagic acid (Saha et al., 2016). Animal experiments using C57BL/6 mice revealed potent anti-inflammatory properties of urolithin A, as it efficiently reduced phorbol myristate acetate (PMA)-induced mouse ear edema formation (Saha et al., 2016). Urolithin A also lessened myocardial ischemia/reperfusion injury and reduced myocardial infarct size in mice via the phosphoinositide 3-kinase/Akt (PI3K/Akt) pathway (Tang et al., 2017).

Altogether, the studies presented above pointed to beneficial vascular effects of urolithins and especially metabolite urolithin A. Additional information on metabolic fate and health effects of ellagitanins and urolithins can be found in several recent reviews (Garcia-Muñoz and Vaillant, 2014; Lipinska et al., 2014; Landete et al., 2016; Tomas-Barberan et al., 2017).

In addition to the above presented effects of hydrolyzable tannins (ellagitannins and gallotannins), their derivative ellagic acid, or their common metabolites urolithins, but also other substances were shown to contribute to beneficial effects of pomegranate products. These include (poly)phenolic compounds anthocyanins (Alighourchi et al., 2008; Fischer et al., 2011a) and flavonoids (Sudheesh and Vijayalakshmi, 2005; Ricci et al., 2006), as well as fatty acids (Kaufman and Wiesman, 2007). For example, anthocyanins exhibit anti-inflammatory activities (Vendrame and Klimis-Zacas, 2015). Flavonoid naringin abundantly present in pomegranate juice (Mphahlele et al., 2014) is considered to contribute (together with flavonoid naritutin) to the anti-hypertensive effects of sweetie juice in humans (Reshef et al., 2005). Puninic acid was shown to ameliorate glucose tolerance and diminish obesity-related inflammation via an activation of PPAR-γ and α (Hontecillas et al., 2009). Quercetin present in juice, seed, and peel of pomegranate (Artik, 1998; Borges and Crozier, 2012; Ambigaipalan et al., 2016) is known to mediate endothelium-dependent vasodilatation via stimulation of both the NO/cyclic guanylyl monophosphate (cGMP) pathway and endothelium-derived hyperpolarizing factor (EDHF) (Khoo et al., 2010).

## Clinical studies on pomegranate in the context of cardiovascular diseases

Many clinical studies investigating the effects of pomegranate in the context of CVDs were performed in the last two decades. These works profusely demonstrated the vasculoprotective properties of pomegranate products (Table 4). Nevertheless, some of these studies pointed to the fact that when applying pomegranate for a longer period or in high amounts, certain possible side effects of such treatment (mainly diarrhea) might occur (Paller et al., 2013).

**Table 4:** Outcome of clinical studies involving intake of pomegranate juice or peel hydro alcoholic extract.

**Type of the study/Number of probands**	**Clinical outcome**	**References**
Daily consumption of pomegranate juice for 2 weeks by hypertensive patients (*N* = 10)	Reduction in ACE activity by 36% and of a systolic blood pressure by 5%	Aviram and Dornfeld, 2001
A long-duration intake of pomegranate juice (for 3 years) by patients with carotid artery stenosis (*N* = 19)	Reduction in systolic blood pressure by 12%, decrease in common carotid intima-media thickness up to 30%	Aviram et al., 2004
A 4-week consumption of pomegranate juice by healthy women (*N* = 51)	A mild, but significant reduction in blood pressure (without significantly changing serum ACE activity)	Lynn et al., 2012
Intake of pomegranate juice by hypersensitive men (*N* = 13)	Decrease in blood pressure while other parameters (serum concentrations of CRP, E-selectin, VCAM-1, ICAM-1, and IL-6) remain unchanged	Asgary et al., 2013
Consumption of pomegranate juice by hypertensive patients (*N* = 21)	Significant reduction in systolic as well as diastolic blood pressure	Asgary et al., 2014
Intake of pomegranate peel hydro alcoholic extract by obese women with dyslipidemia (*N* = 38)	Significant reduction in systolic blood pressure	Haghighian et al., 2016
A meta-analysis focusing on effects of pomegranate consumption on CRP	No significant correlation between pomegranate consumption and CRP levels	Sahebkar et al., 2016
A meta-analysis focusing on blood pressure lowering effects of intake of pomegranate juice	Decrease in systolic blood pressure levels (regardless of the duration and dose of the juice consumed in the evaluated studies). A borderline significant effect in reducing of diastolic blood pressure by doses higher than 240 cc (eight ounces)	Sahebkar et al., 2017

In hypertensive patients, daily consumption of pomegranate juice for 2 weeks reduced the activity of ACE by 36% as well as diminished systolic blood pressure by 5% (Aviram and Dornfeld, 2001). The same group also reported that a long-duration intake of pomegranate juice (for 3 years) by patients with carotid artery stenosis significantly reduced their blood pressure, LDL oxidation and common carotid intima-media thickness (Aviram et al., 2004). A 4-week consumption of pomegranate juice reduced significantly blood pressure in a cohort of 51 healthy women (without significantly changing serum ACE activity; Lynn et al., 2012). Another study involving 13 hypersensitive men demonstrated that intake of pomegranate juice lowered blood pressure (Asgary et al., 2013). However, in these patients the levels of some clinical parameters, such as serum concentrations of C-reactive protein (CRP), E-selectin, VCAM-1, ICAM-1, and IL-6 remained unchanged (Asgary et al., 2013). A subsequent study involving 21 hypertensive patients showed that consumption of pomegranate juice significantly reduced systolic as well as diastolic blood pressure (Asgary et al., 2014). In addition, a double blind, randomized, placebo controlled pilot study revealed that the pomegranate peel hydroalcoholic extract reduced cardiovascular risk factors in obese women with dyslipidemia (Haghighian et al., 2016).

Although a meta-analysis evaluating the effects of pomegranate consumption on CRP concentrations did not reveal a significant correlation between these parameters (Sahebkar et al., 2016), the effects of pomegranate consumption on blood pressure regulation in accomplished animal and human studies seem to be clinically relevant. In a recent review it was concluded that both pomegranate juice and seed oil can effectively lower blood pressure (Asgary et al., 2017). Another recently accomplished meta-analysis came to the same conclusions, as intake of pomegranate juice decreased levels of systolic blood pressure regardless of the duration and dose of the juice consumed in the evaluated studies, whereas doses more than 240 cc (eight ounces) exhibited a borderline significant effect in reducing of a diastolic blood pressure (Sahebkar et al., 2017). The authors of this meta-analysis determined a constant benefit of pomegranate juice intake on blood pressure, which may be considered clinically relevant. Additional information of how pomegranate affects vasculature can be found in some other reviews (Lansky and Newman, 2007; Aviram and Rosenblat, 2013; Hyson, 2015; Zheng et al., 2017). In addition to many described beneficial effects of pomegranete on endothelial function, pomegranate juice was also found to enhance the inhibitory effect of NO on vascular smooth muscle cell proliferation (Ignarro et al., 2006). This aspect might be clinically relevant and a subject of further studies, as vascular smooth muscle cell proliferation plays an important role in the development and progression of atherosclerosis and restenosis (Uhrin et al., 2018; Wang et al., 2018).

## Conclusion

Pomegranate, an ancient and highly distinctive fruit, is a rich source of natural bioactive constituents. Various studies showed that pomegranate and its products exhibit protective effects on the cardiovascular system. These vasculoprotective effects include diminishing of oxidative stress, positive influencing macrophage-, endothelial cell-, and platelet function, lowering lipid oxidation, reducing blood glucose levels, vasodilatory effects as well as decreasing blood pressure via an inhibition of ACE activity. In light of the altogether promising outcome of numerous pre-clinical and clinical studies, pomegranate is advocated to be used as a dietary supplement for prevention and treatment of cardiovascular diseases, thus representing a supplementary non-pharmacological therapy for cardiovascular diseases.

## Author contributions

DW, CÖ, IA-R, SC, JP, PU, and AA wrote the first draft of the manuscript. JH and AJ prepared Tables 2–4 during the revision and NT improved the revised version of the manuscript.

### Conflict of interest statement

The authors declare that the research was conducted in the absence of any commercial or financial relationships that could be construed as a potential conflict of interest.
